# Retargeting of NK-92 Cells against High-Risk Rhabdomyosarcomas by Means of an ERBB2 (HER2/Neu)-Specific Chimeric Antigen Receptor

**DOI:** 10.3390/cancers13061443

**Published:** 2021-03-22

**Authors:** Leonie D. H. Gossel, Catrin Heim, Lisa-Marie Pfeffermann, Laura M. Moser, Halvard B. Bönig, Thomas E. Klingebiel, Peter Bader, Winfried S. Wels, Michael Merker, Eva Rettinger

**Affiliations:** 1 Department for Children and Adolescents, Division for Stem Cell Transplantation, Immunology and Intensive Care Medicine, University Hospital Frankfurt, Goethe University, 60590 Frankfurt am Main, Germany; leonie.gossel@kgu.de (L.D.H.G.); catrin.heim@kgu.de (C.H.); laura.moser@kgu.de (L.M.M.); peter.bader@kgu.de (P.B.); michael.merker@kgu.de (M.M.); 2 Department of Cellular Therapeutics/Cell Processing, Institute for Transfusion Medicine and Immunohematology Frankfurt am Main, Goethe University Medical School, 60528 Frankfurt am Main, Germany; l.pfeffermann@blutspende.de (L.-M.P.); h.boenig@blutspende.de (H.B.B.); 3 German Cancer Consortium (DKTK), Partner Site Frankfurt/Mainz, 60590 Frankfurt am Main, Germany; thomas.klingebiel@kgu.de (T.E.K.); wels@gsh.uni-frankfurt.de (W.S.W.); 4 Frankfurt Cancer Institute (FCI), 60596 Frankfurt am Main, Germany; 5 Universitäres Centrum für Tumorerkrankungen (UCT), 60590 Frankfurt am Main, Germany; 6 Department of Medicine, Division of Hematology, University of Washington, Seattle, WA 98198-7720, USA; 7 Department for Children and Adolescents, University Hospital Frankfurt, Goethe University, 60590 Frankfurt am Main, Germany; 8 Georg-Speyer-Haus, Institute for Tumor Biology and Experimental Therapy, 60596 Frankfurt am Main, Germany

**Keywords:** RMS, ERBB2, HER2/neu, NK-92, CAR, cancer immunotherapy

## Abstract

**Simple Summary:**

In this study, we apply the ERBB2-chimeric antigen receptor (CAR)-modified natural killer (NK) cell line NK-92 (NK-92/5.28.z), a well-defined, good manufacturing practice (GMP)-compliant, third-party, off-the-shelf immune effector cell product as a novel immunotherapeutic approach for the treatment of high-risk rhabdomyosarcomas. Our preclinical in vitro data show enormous potential to improve immunotherapy of ERBB2-positive high-risk rhabdomyosarcoma a still incurable, rapidly lethal disease, assigning to NK-92/5.28.z cells rather than to unmodified parental NK-92 cells a multifarious role as ERBB2-specific CAR-targeted killers and modulators of endogenous adaptive immunity of the host, justifying the further evaluation of this approach in in vivo mouse xenograft models as a prerequisite for a possible future phase I/II clinical trial in defined subsets of high-risk rhabdomyosarcoma patients.

**Abstract:**

The dismal prognosis of pediatric and young adult patients with high-risk rhabdomyosarcoma (RMS) underscores the need for novel treatment options for this patient group. In previous studies, the tumor-associated surface antigen ERBB2 (HER2/neu) was identified as targetable in high-risk RMS. As a proof of concept, in this study, a novel treatment approach against RMS tumors using a genetically modified natural killer (NK)-92 cell line (NK-92/5.28.z) as an off-the-shelf ERBB2-chimeric antigen receptor (CAR)-engineered cell product was preclinically explored. In cytotoxicity assays, NK-92/5.28.z cells specifically recognized and efficiently eliminated RMS cell suspensions, tumor cell monolayers, and 3D tumor spheroids via the ERBB2-CAR even at effector-to-target ratios as low as 1:1. In contrast to unmodified parental NK-92 cells, which failed to lyse RMS cells, NK-92/5.28.z cells proliferated and became further activated through contact with ERBB2-positive tumor cells. Furthermore, high amounts of effector molecules, such as proinflammatory and antitumoral cytokines, were found in cocultures of NK-92/5.28.z cells with tumor cells. Taken together, our data suggest the enormous potential of this approach for improving the immunotherapy of treatment-resistant tumors, revealing the dual role of NK-92/5.28.z cells as CAR-targeted killers and modulators of endogenous adaptive immunity even in the inhibitory tumor microenvironment of high-risk RMS.

## 1. Introduction

Rhabdomyosarcoma (RMS) is the most common soft-tissue sarcoma in children and accounts for 5% of all pediatric cancers [1,2]. The five-year overall survival (OS) rates vary from 78% in low-risk patients to a dismal 8% in high-risk patients [3].

Unfortunately, most of the affected children (56%) have more than one risk factor and thus a dismal prognosis [3,4]: patients at an age greater than 10 years and patients with primary tumors in the extremities, trunk, retroperitoneum, or parameningeal region appear to have a particularly poor outcome [3,5]. In addition to older age and unfavorable primary tumor site, alveolar histopathology also has a negative influence on patient outcome, as alveolar RMS (aRMS) has a higher risk of metastatic disease than other types of RMS, with 68% of patients already carrying metastases when they are first diagnosed with aRMS [3,6,7]. Other unfavorable prognostic factors are bone or bone marrow (BM) involvement, regional lymph node involvement, the presence of multiple metastases, large tumor size, invasion, and a positive fusion status by histopathology [2,3,8,9,10].

As such, high-risk RMS needs to be recognized as a systemic cancer and must be treated accordingly [11]. However, current treatment strategies such as surgical resection, radiation therapy, and systemic chemotherapy with vincristine, irinotecan, doxorubicin, cyclophosphamide, ifosfamide, and etoposide do not provide lasting therapeutic benefits [4,12,13]. On the one hand, treatment-related toxicities are considerable, particularly in the high-risk group, where patients are more susceptible to adverse effects and suffer from cumulative toxicities [14,15]. On the other hand, the relapse rate for high-risk RMS is high, and the prognosis is low in relapsed disease [3,8,16,17]. Previous attempts to extend survival by intensifying and broadening chemotherapy or by offering haploidentical stem cell transplantation and new agents such as cixutumumab, a monoclonal antibody (mAb) directed against the human insulin-like growth factor-1 receptor (IGF-1R), or temozolomide have not improved outcomes, and some of these attempts have merely added to the known toxicities [4,9,17,18,19].

Accordingly, novel tumor-specific and less toxic therapies are desperately needed for high-risk RMS patients [4], especially for those who are older than 10 years of age, with an alveolar histopathology subtype, with unfavorable primary tumor sites, and with metastatic disease with bone or BM involvement [3].

The clinically usable established human natural killer (NK) cell line NK-92 is capable of directly eliminating cancer cells and indirectly triggering subsequent adaptive antitumor immune responses [20,21]. NK-92 cells lack the expression of most inhibitory NK cell receptors but possess a full repertoire of activating NK cell receptors such as natural cytotoxicity receptors (NCRs) and the C-type lectin-like receptor NKG2D [22,23]. Unlike primary NK cells, the NK-92 cell line can be continuously expanded in vitro in interleukin (IL)-2-containing medium into a good manufacturing practice (GMP)-compliant product with clinically relevant cell doses [22,24,25]. Furthermore, early-phase trials have confirmed the clinical safety of infusions of irradiated NK-92 cells, which have resulted in durable responses in some treated patients despite the limited persistence of the irradiated cells [22,26]. Therefore, NK-92 cells can be administered to human leukocyte antigen (HLA)-mismatched recipients as an off-the-shelf product, as NK-92 cell therapy does not cause graft versus host disease (GVHD) and its antitumor effects are not major histocompatibility complex (MHC)-restricted [21,26].

The cytotoxic activity of NK-92 cells and their tumor specificity can be further enhanced by genetic engineering, enabling them to express an integrated chimeric antigen receptor (CAR) that targets a tumor-associated antigen (TAA) of choice [27,28]. As has been demonstrated for different types of CAR-engineered immune effector cells, CARs can respond to levels of TAAs that are too low to trigger antibody-dependent cell-mediated cytotoxicity (ADCC) upon application of therapeutic mAbs [15,29,30,31,32]. Furthermore, in immunocompetent animal models, in addition to their direct cytotoxicity, CAR-NK-92 cells have been shown to trigger endogenous adaptive immune responses against tumors [21,28]. This effect, together with their exquisite cytotoxic potential and specific homing capabilities, could make CAR-NK-92 cells a promising approach for treating disseminated solid tumors, such as high-risk RMS.

The choice of the TAA is of vital importance for CAR-based strategies when considering potential on-target/off-tumor effects directed against healthy tissues also expressing the targeted antigen [27,33]. ERBB2 is a growth factor receptor expressed at moderate levels by many epithelial tissues but is overexpressed by different types of solid tumors, in which it contributes to malignant transformation [34]. ERBB2 expression was reported first for RMS cell lines, including cell lines with alveolar histotype by Ricci and colleagues in 2000 [35]. ERBB2 was more systematically analyzed in a sizable subset of childhood rhabdomyosarcoma tumors by Ganti et al. in 2006 [36] and in an even larger tumor series of 105 adult RMS patients by Armistead et al. in 2007 [37]. Altogether, ERBB2 appears to be more prevalent in children and in the alveolar histopathology subtype [15,36]. The safety of treatment with ERBB2-specific autologous CAR-T cells was initially demonstrated in a phase I/II clinical trial in sarcoma patients at Baylor College of Medicine, Houston, Texas, USA [38]. More recently, the same group reported durable remission over four years after initiating ERBB2-CAR-T cell infusions combined with checkpoint inhibition in a child with ERBB2 high-level surface expression of high-risk alveolar RMS [30]. ERBB2 mutation, which is found in 1.4% of RMS tumors without a PAX gene fusion, can play a causal role for pathway alterations in a subset of RMS tumors [39], as Nanni et al. showed that activation of the ERBB2 oncogene led to the onset of RMS in transgenic p53 mutant mice [40]. Therefore, ERBB2 is an important target for CAR therapy.

By extending this approach to NK cells, an ERBB2-specific NK-92 cell line equipped with a second-generation CAR very similar to the CAR previously reported by Ahmed and colleagues [38] was generated by GMP-compliant lentiviral transduction [21,25,41]. These NK-92/5.28.z cells displayed high and specific activity against ERBB2-positive cancer cells, serial target cell killing, and homing to distant tumor sites in preclinical models of cancers of different solid tumor origins [28,41,42]. Even if kept under hypoxic conditions or in the presence of elevated transforming growth factor (TGF)-β concentrations, NK-92/5.28.z cells retained their specific cytotoxicity and functionality, suggesting that they may be able to overcome the inhibitory tumor microenvironment (TME) of solid tumors in vivo to a certain extent [21,28], which is considered a severe challenge for CAR-T cells [33]. NK-92/5.28.z cells are also expected to have a much lower risk of inducing cytokine-release syndrome (CRS) than CAR-T cells, as the cytotoxic cytokine and chemokine profiles are different between CAR-NK-92 and CAR-T cells [23,28,33]. Currently, the NK-92/5.28.z cell line is being tested in a phase I clinical trial for the treatment of recurrent ERBB2-positive glioblastoma [21], and the results could aid in extending this approach to other disease entities.

As the first step in this direction, we investigated the efficacy of NK-92/5.28.z cells against aRMS cells in vitro to evaluate their potential as a novel treatment strategy for high-risk RMS.

## 2. Materials and Methods

### 2.1. Cell Lines and Cell Culture

**Figure 1:**
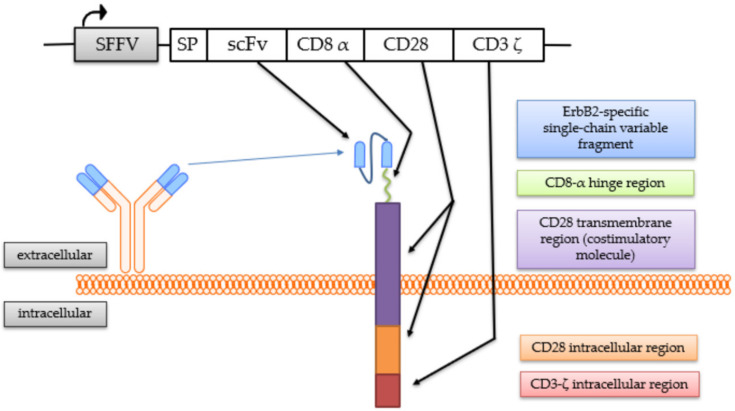
Chimeric antigen receptor (CAR) expressed by NK-92/5.28.z cells. The coding regions of the lentiviral CAR vector and the CAR molecule with its protein domains are schematically shown.

The generation of the continuously expanding effector cell line NK-92/5.28.z by transduction with a lentiviral CAR vector has been described previously [25,41]. These cells stably express a second-generation CAR consisting of an ERBB2-specific scFv antibody fragment derived from the antibody FRP5 [43] with a modified CD8α hinge region and a CD28 transmembrane and intracellular domain as a costimulatory molecule, as well as a CD3ζ intracellular domain [41] (Figure 1).

NK-92/5.28.z cells were cultured in X-Vivo 10 (Lonza, Basel, Switzerland) supplemented with 5% fresh frozen human plasma of blood type AB (DRK-Blutspendedienst, Frankfurt am Main, Germany) and 100 IU IL-2/mL (Proleukin^®^ S; Novartis, Nurnberg, Germany) [25]. The NK-92 cell line, which is derived from a human NK cell non-Hodgkin’s lymphoma (kindly provided by Prof. Hans G. Klingemann), was cultured under the same conditions as the NK-92/5.28.z cells. The aRMS cell lines RH30 and RH41 were cultured in Roswell Park Memorial Institute medium (Gibco RPMI Medium 1640 (1X) + GlutaMAX™, Thermo Scientific, Waltham, MA, USA) supplemented with 10% fetal bovine serum (FBS, low in endotoxin; Sigma-Aldrich, St. Louis, MO, USA). RMS cell lines were obtained from the DSMZ (Deutsche Sammlung von Mikroorganismen und Zellkulturen GmbH, Braunschweig, Germany). Primary aRMS cells (kindly provided by Sibylle Wehner, University Hospital Frankfurt am Main, Laboratory of Pediatric Hematology, Frankfurt am Main, Germany) were obtained after written and informed consent of the patient and the patient’s parents and cultured according to the RH30 and RH41 cell protocols. The ERBB2-positive and ERBB2-negative human breast carcinoma cell lines MDA-MB-453 and MDA-MB-468 (ATCC; Manassas, VA, USA) were cultured in Dulbecco’s modified Eagle’s medium (DMEM; 1gGluc m.Glutamax/Nabic o.Pyr, Gibco^®^; Life Technologies, Darmstadt, Germany) supplemented with 10% FBS [41]. All cell lines were incubated in a humidified atmosphere at 37 °C and 5% CO_2_ and kept under antibiotic-free conditions.

### 2.2. Cell Surface Staining and Flow Cytometric Analysis for the Characterization of Effector and Target Cells

Different cell lines were analyzed for various cell surface markers using a BD FACSCanto II flow cytometer (BD Biosciences, San Jose, CA, USA). Monoclonal antibodies were conjugated herein with phycoerythrin (PE) or allophycocyanin (APC).

aRMS cell lines RH30 and RH41, as well as primary aRMS cells, were tested for ERBB2 expression by staining with an anti-CD340 antibody (anti-CD340, ERBB2/HER-2, PE, BioLegend, London, UK) using the human breast carcinoma cell lines MDA-MB-453 and MDA-MB-468 as positive and negative controls, respectively [41], as well as an isotype control to substantiate the result (IgG1κ Isotype Ctrl FC Antibody, PE, BioLegend, London, UK). In parallel, aRMS cells were assessed for MIC-A/-B (MICA/MICB Antibody, APC, BioLegend, London, UK; isotype control, IgG2aκ Isotype Ctrl FC Antibody, APC, BioLegend, London, UK), as well as for ULBP-1 (ULBP-1 Antibody, APC, Miltenyi Biotec, Bergisch Gladbach, Germany; isotype control, IgG2a Isotype Ctrl FC Antibody, APC, Miltenyi Biotec, Bergisch Gladbach, Germany).

The effector cell line NK-92/5.28.z was analyzed for ERBB2-CAR expression using an ERBB2-IgG-Fc chimera (Sino Biological Inc., Beijing, China) after nonspecific Fc receptor blocking (Human TruStain FcX™, BioLegend, London, UK) and incubation on ice for 20 min. To visualize the chimera, a secondary anti-IgG-Fc monoclonal antibody conjugated with APC (BioLegend, London, UK) was used. Parental NK-92 cells were included as a control.

Cell surface staining was performed according to the manufacturers’ instructions. Aliquots of 1.5 × 10^6^ cells per cell line per FACS^®^ tube (BD Biosciences, San Jose, CA, USA) were washed twice in phosphate-buffered saline (PBS Dulbecco, Gibco^®^, Life Technologies, Darmstadt, Germany) followed by 15 min of incubation with an antibody conjugated to a fluorochrome. The cells were then washed again, and the data for 5–10 × 10^4^ events per tube were acquired by flow cytometry using FACSDiva software (Version 6.1.3, BD Biosciences). The gates were set on viable cells, which were distinguished according to the absence or expression of the marker in question. Kaluza Analysis 3.1 (Beckman Coulter, Krefeld, Germany) was used for data analysis. Single data are given as median fluorescence intensity (MFI) or percentage of gated cells but are given as mean ± SD in case of replicated data.

### 2.3. Short-Term Cytotoxic Potential of NK-92/5.28.z and Parental NK-92 Cells against aRMS Tumor Cell Suspensions

To analyze the short-term cytotoxicity of cells against aRMS tumor cell suspensions, the europium release assay was used, which is based on bis (acetoxymethyl) 2,2′:6′,2″- terpyridine- 6,6″- dicarboxylate (BATDA) infiltrating the target cell membrane. After the ester bond of the compound is hydrolyzed, hydrophobic 2,2′:6′,2″ -terpyridine-6,6″ -dicarboxylic acid (TDA) is trapped inside the intact cell. If a cell is lysed by an effector cell, TDA will be released, and as it binds to europium, it builds a stable fluorescent chelate complex. The measured fluorescent signal correlates directly with the proportion of lysed target cells (PerkinElmer Inc. Delfia EuTDA Cytotoxicity Reagents).

In brief, target cells were adjusted to 2 × 10^6^/mL, labeled with 5 µL BATDA reagent (PerkinElmer, Waltham, MA, USA), and incubated on a shaker (Shaker SI100; Pharmacia Diagnostics, Uppsala, Sweden) for one hour. To lower the background emission, the cells were then washed four times. In the case of aRMS and human breast carcinoma cells, probenecid (Sigma-Aldrich, St. Louis, MO, USA) was added to prevent BATDA from being transported out of the intact cell by multidrug transporters, thus altering the results. Afterward, the cell densities of both the target and effector cells were adjusted to 5 × 10^4^/mL and 2 × 10^6^/mL, respectively. Cocultures were loaded into a round-bottom plate (96-well plate Nunclon™; Thermo Scientific, Waltham, MA, USA) at effector-to-target (E:T) ratios of 40:1, 20:1, 10:1, and 5:1. Target cells incubated alone were used to determine spontaneous release, and target cells incubated in the presence of 20% Triton™ X-100 solution (Sigma-Aldrich, St. Louis, USA) were used to determine the maximum lysis value. After another incubation period of three hours, the supernatants were transferred into a flat-bottom plate (96-well plate Falcon^®^; Corning, New York, NA, USA), and europium solution (Delfia^®^, PerkinElmer, Waltham, MA, USA) was subsequently added. The emitted signals were measured using a Fluorometer Victor 3™ 1420 (PerkinElmer, Waltham, MA, USA), and specific lysis of the target cells was calculated as follows: specific lysis (%) = 100 × (experimental release (counts) − spontaneous release (counts))/(maximum release (counts) − spontaneous release (counts)) [44].

### 2.4. Long-Term Cytotoxicity Assessment of NK-92/5.28.z and Parental NK-92 Cells against aRMS Monolayers

To determine the cytotoxic capacity over a longer time span, a 16-h coculture assay was performed using a Celigo cell cytometer (Nexcelom Bioscience LLC., Lawrence, MA, USA), which enabled assessment of direct cell counts by bright field imaging, as previously described by Merker et al. [15]. In brief, cells at a target density of 2 × 10^5^ per mL per well were plated in a 12-well plate (Falcon^®^, flat bottom, Corning, New York, NA, USA). During a six-hour incubation period, the cells were given time to adhere. The supernatants were removed; the cells were washed once in 1 mL PBS, and 0.5 mL culture medium was added. The remaining cells were counted using a Celigo cell cytometer before the addition of effector cells at E:T ratios of 10:1, 5:1, and 1:1, while target cells incubated without effector cells served as controls. Cells were coincubated for 16 h. Thereafter, the supernatants, including the effector cells in suspension, were removed, and the cells were washed twice in PBS. Residual cells were counted again by a Celigo cell cytometer. The specific lysis was calculated for each E:T ratio as follows: specific lysis (%) = (1 − mean of the remaining target cells)/mean of the corresponding negative control [15].

### 2.5. Analysis of Target Cell Killing of NK-92/5.28.z and Parental NK-92 Cells by Time-Lapse Microscopy

To not only determine the cytotoxic capacity of NK-92/5.28.z cells at a given time point but also follow the kinetics of the killing process, cocultures of target and effector cells were monitored over a period of 18 h. Target cells (1 × 10^6^) were placed on a petri dish (35 × 10 mm, Nunclon™ Delta; Thermo Fisher Scientific, Waltham, MA, USA) and given a six-hour incubation period to adhere. After gently removing the supernatant, effector cells were added at an E:T ratio of less than 1:1, and cocultures were placed under a microscope (Olympus IX71, Research Inverted System Microscope) for video analysis using a 6.3 × magnification. Target and effector cells were discriminated by their different microscopic morphology described in the results (Section 3.1) and discussion section as well as by their different culture behaviors growing as adherent or suspension cells, respectively Figure S1. Viable adherent target cells remained attached to the bottom of the respective culture tissues while effector cells floated and grew suspended in the culture. Killed target cells lost their growth pattern and floated as cell debris in the culture as well. 18 h later, suspension cells were gently removed, and numbers of effector cells were counted using trypan blue staining (Sigma-Aldrich, St. Louis, MO, USA). Then, the remaining tumor cells were chemically removed, and cell numbers were counted.

### 2.6. Cytotoxic Activity of NK-92/5.28.z and Parental NK-92 Cells against aRMS Tumor Spheroids

Three-dimensional tumor spheroids were generated using an ultralow attachment 96-well round-bottom plate without additional coating (Corning Incorporated, Corning, NU, USA), and 5 × 10^3^ target cells in 200 µL medium were plated in each well. The plate was then centrifuged for 10 min (1000× *g*). Half of the culture medium without supplements (100 µL) was replaced every three to four days.

On day four of cell culture, 2 × 10^5^ or 0.5 × 10^5^ effector cells were added to each well. Images were taken every day using a Celigo cell cytometer (with the embryoid body application), starting six hours after the beginning of coculture and ending on day 10. Discrimination of target and effector cells was shown by their different growing behaviors as tumor spheroids and immune effector cell clones/clusters.

### 2.7. Confirmation of ERBB2 Expression on Tumor Spheroid Cells

The expression of ERBB2 on the tumor cells within the spheroids was investigated by gently resuspending the spheroids, followed by staining with the anti-CD340 antibody and analysis with a flow cytometer using the same protocol as described above (see Section 2.2 Cell Surface Staining and Flow Cytometric Analysis for the Characterization of Effector and Target Cells). Tumor spheroids were harvested on days 1, 3, 5, 7, 10, and 14 of culture for assessment of ERBB2. The results were compared to those of isotype controls and the ERBB2 expression levels of MDA-MB-453 and MDA-MB-468 cells, which were used as positive and negative controls growing in regular culture, respectively.

### 2.8. Quantitative Analysis of Immunoregulatory Factors Secreted by NK-92/5.28.z and Parental NK-92 Cells in Coculture with aRMS Cells

A bead-based immunoassay using the Multi-Analyte Flow Assay Kit LEGENDplex™ (Human CS8/NK Panel, 13-plex, Cat. No. 740267; BioLegend, San Diego, CA, USA) was performed according to the manufacturer’s instructions to detect the levels of IL-2, IL-4, IL-6, IL-10, IL-17A, interferon (IFN)-γ, tumor necrosis factor (TNF)-α, soluble Fas and Fas ligand (FasL), granzymes A and B, granulysin, and perforin secreted by NK-92/5.28.z and NK-92 cells while they were in contact with tumor cells. The assay uses capture beads that are conjugated to specific antibodies that bind to the target analytes described above. After washing, biotinylated detection antibodies were added, followed by the addition of streptavidin-phycoerythrin (SA-PE). SA-PE binds to the detection antibodies. In these assays, the fluorescent signal intensities measured with a BD FACS Canto™ flow cytometer directly correlate to the quantity of analytes bound. Additionally, standard curves were generated to ascertain the concentrations of the particular analytes.

To assess the responses of NK-92/5.28.z and NK-92 cells while attacking aRMS cells, supernatants of the europium release assay cultures at E:T ratios of 20:1 (absolute cell counts per well, 1 × 10^5^ effector cells and 5 × 10^3^ target cells) after three hours of coculture were collected and preserved at −80 °C until the immunoassay was performed.

The standard preparation and the assay were both performed according to the information provided by the manufacturer. Evaluation of the immunoassay was performed using LEGENDplex™ Data Analysis Software (V 7.0) (VigeneTech, Carlisle, PA, USA).

### 2.9. Analysis of Degranulation by CD107a Staining

The CD107a staining was performed as described elsewhere [45]. In short, 1 × 10^5^ RH30 cells were coincubated with the same number of NK-92/5.28.z or NK-92 cells (E:T ratio 1:1) for two hours at 37 °C. For the basal CD107a expression, the effector cells were incubated under the same conditions without RH30 target cells. Thereafter, cell suspensions were washed once with PBS and stained with CD107a-FITC and CD56-PE/Cy7 antibodies (BioLegend, London, UK). Measurements were conducted by BD FACSCanto10c (BD Biosciences, San Jose, USA), and gates were set on CD107a-CD56 double-positive singlets identifying them as degranulating NK-92/5.28.z or NK-92 cells by FlowJo Software 10.3 (BD Bioscience, Ashland, OR, USA).

### 2.10. Statistical Analysis

For statistical analysis and graphical representation of data, GraphPad Prism 6.0 software (La Jolla, CA, USA) was used. The results are given as the mean ± SD. A two-tailed Student’s t-test was used to evaluate the differences between values. Differences were considered significant for *p* < 0.05 (*), *p* < 0.01 (**), *p* < 0.005 (***), and *p* < 0.0001 (****). Flow cytometry data are given as MFI or percentage of gated cells but are given as mean ± SD in case of replicated data.

## 3. Results

### 3.1. Alveolar Rhabdomyosarcoma Cell Characterization—ERBB2 Is a Targetable TAA

**Figure 2:**
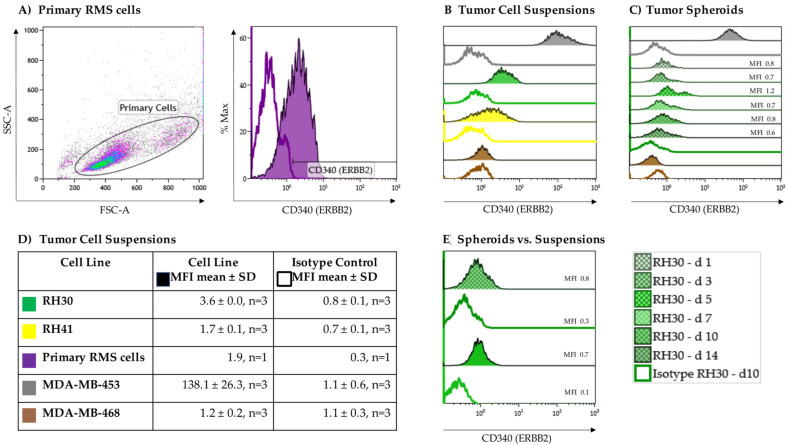
ERBB2 expression on alveolar rhabdomyosarcoma (aRMS) cells. RH30, RH41, and (**A**) primary tumor cell suspensions showed different variability in size (forward (FSC) vs. side scatter (SSC)) and displayed low but homogenous ERBB2 expression compared to isotype controls. The MDA-MB-453 and MDA-MB-468 breast cancer cell lines served as positive and negative controls, respectively (**B**,**D**). Compared to tumor cell suspensions stable ERBB2 expression was detectable on tumor spheroids during tumor growth (**C**,**E**).

As a proof of concept, the expression of the growth factor receptor ERBB2 on the aRMS cell lines RH30 and RH41 grown in suspension cultures was verified via flow cytometry using an isotype control as a negative control (Figure 2B,D). To substantiate the results, primary RMS cells from a BM aspirate of, up to that point, an untreated, newly diagnosed adolescent patient with alveolar histopathologic subtype RMS positive for the PAX3-FOXO1 fusion gene were also analyzed for surface expression of ERBB2 (Figure 2A). The heterogeneity in cell size and morphology shown by forward (FSC) versus side scatter (SSC) is a typical feature of RMS. All aRMS tumor cell suspensions displayed low but homogenous ERBB2 expression (RH30, MFI 3.6 ± 0.0, *n* = 3; RH41, MFI 1.7 ± 0.1, *n* = 3; primary aRMS cells, MFI 1.9, *n* = 1) (Figure 2D).

To evaluate whether ERBB2 is downregulated in the process of tumor growth, ERBB2 expression was assessed on RH30 cell suspensions becoming tumor spheroids over a 14-day period (Figure 2C). The ERBB2-negative MDA-MB-468 breast cancer cell line was used as a negative control (MDA-MB-468, MFI 1.2 ± 0.2, *n* = 3), while the MDA-MB-453 breast cancer cell line served as a positive control (MDA-MB-453, MFI 138.1 ± 26.3, *n* = 3). Overall, stable expression of ERBB2 was documented after 10 days of culture (Figure 2E).

Considering the potential impact of intrinsic NK-92-mediated tumor cell lysis, the NKG2D ligands MHC class I chain-related proteins A and B (MIC-A/-B) and UL16 binding protein 1 (ULBP-1) were assessed. MIC-A/-B showed no and ULBP-1 very low but consistent expression on RH30 cells (ULBP-1, MFI 0.9 ± 0.0, *n* = 3).

### 3.2. Effector Cell Characterization

The expression of the ERBB2-specific second-generation CAR on NK-92/5.28.z cells was assessed via flow cytometry using a recombinant ERBB2-IgG-Fc fusion protein for detection of functional CAR molecules. Stable CAR expression was confirmed via comparisons with unmodified parental NK-92 cells (Figure S2).

### 3.3. Cytotoxic Potential of NK-92/5.28.z and Parental NK-92 Cells against ARMS Tumor Cell Suspensions

In a 3-h coincubation europium release assay, NK-92/5.28.z cell-mediated lysis of tumor cell suspensions was directly compared to that of unmodified parental NK-92 cells. ERBB2-negative MDA-MB-468 cells were used as a negative control, and ERBB2-overexpressing MDA-MB-453 cells were used as a positive control.

**Figure 3:**
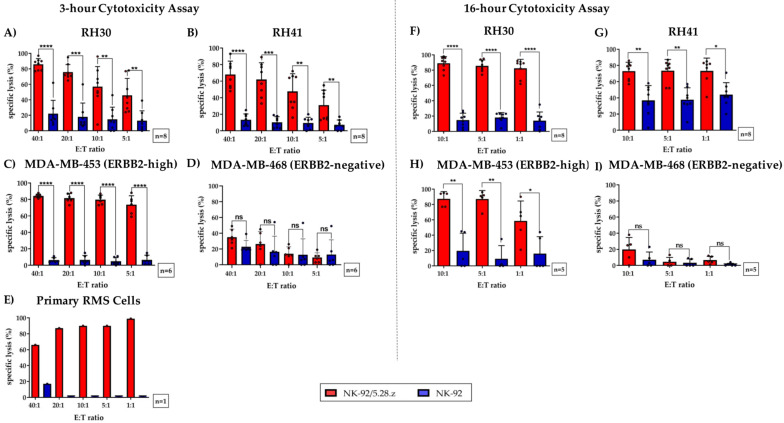
Specific cytotoxicity of NK-92/5.28.z cells against aRMS cell lines growing in suspension or as tumor cell monolayers. NK-92/5.28.z cells were compared with unmodified parental NK-92 cells in a 3-h cytotoxicity assay against cell suspensions of (**A**) RH30 cells, (**B**) RH41 cells, (**E**) primary aRMS cells, (**C**) MDA-MB-453 cells (which served as a positive control), and (**D**) MDA-MB-468 cells (which were used as a negative control). The effector-to-target (E:T) ratios ranged from 40:1 to 5:1 or from 40:1 to 1:1 (in primary RMS cells). Tumor cell lysis was ERBB2-specific and significantly increased when NK-92/5.28.z cells were used, even at low E:T ratios. In a 16-h cytotoxicity assay, the killing capacity of NK-92/5.28.z cells against monolayers of (**F**) RH30, (**G**) RH41, (**H**) MDA-MB-453, and (**I**) MDA-MB-468 tumor cells was assessed in comparison to that of parental NK-92 cells by a Celigo cell cytometer. ARMS monolayers were lysed to a significantly greater extent by NK-92/5.28.z cells than by unmodified parental NK-92 cells. Differences were considered significant for *p* < 0.05 (*), *p* < 0.01 (**), *p* < 0.005 (***), and *p* < 0.0001 (****), or not significant (ns).

At an effector-to-target (E:T) ratio of 40:1, NK-92/5.28.z cells induced a mean specific lysis of 77.4 ± 10.8% (*n* = 8) (Figure 3A), 61.4 ± 14.8% (*n* = 8) (Figure 3B), and 85.0 ± 2.4% (*n* = 6) (Figure 3C) of RH30, RH41, and MDA-MB-453 cells, respectively.

Altogether, NK-92/5.28.z cells demonstrated significantly increased lysis of ERBB2-positive tumor cells compared to NK-92 cells, even at an E:T ratio of 5:1. Of note, ERBB2-specific NK-92/5.28.z cells achieved killing rates of primary aRMS cells of up to 99% (Figure 3E).

As expected, specific lysis of MDA-MB-468 cells (which was used as a negative control) was minimal in all experiments performed (Figure 3D). In addition, the mean specific lysis of MDA-MB-453, RH30, RH41, and primary RMS tumor cells by parental NK-92 cells remained low but varied depending on the number of NK-92 cells used.

### 3.4. Cytotoxic Capacity of NK-92/5.28.z and Parental NK-92 Cells against aRMS Cell Monolayers

To evaluate the capacity of NK-92/5.28.z cells to lyse tumor cell monolayers, we performed a 16-h coincubation cytotoxicity assay using Celigo cell cytometry. As described above (see Section 2.4), RH30, RH41, MDA-MB-453, and MDA-MB-468 cells were used as targets, but in these experiments, they were growing as adherent cell monolayers (Figure 3F–I). At an E:T ratio of 1:1, aRMS cell lines were specifically lysed by NK-92/5.28.z cells, with mean lysis rates of 82.4 ± 11.0% and 73.3 ± 14.7% observed for RH30 and RH41 cells, respectively (Figure 3F,G). In contrast, the degree of tumor cell killing by parental NK-92 cells remained low.

### 3.5. Target Cell Killing by NK-92/5.28.z and Parental NK-92 Cells over 18 Hours

The lysis of tumor cell monolayers was visualized via time-lapse microscopy over an 18-h time period (see the appendix for a movie covering the entire experiment). Therefore, the effects of NK-92/5.28.z and parental NK-92 cells against RH30 tumor cell monolayers were tested in separate experiments, starting with an initial E:T ratio of less than 1:1.

**Figure 4:**
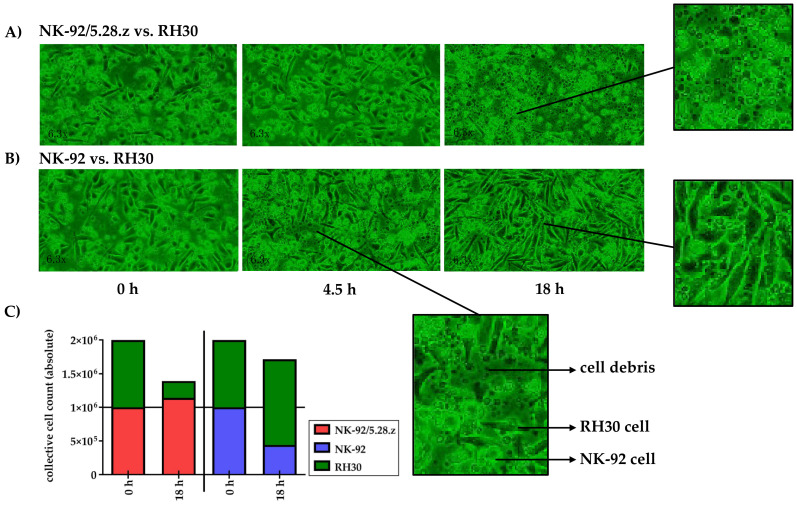
Time-lapse microscopy analysis of NK-92/5.28.z cell activity over 18 h. Images of established RH30 cell monolayers were recorded during coincubation with NK-92/5.28.z (**A**) or parental NK-92 (**B**) cells (*n* = 1). NK-92/5.28.z cells proliferated and almost completely lysed RH30 cell monolayers, while NK-92 cells decreased in number and were not able to lyse RH30 cell monolayers or inhibit tumor growth. The observed effects are shown as exact cell counts (**C**). Here target and effector cells were discriminated by their different microscopic morphology as well as by their different culture behaviors (see Section 2.5).

Target and effector cells were discriminated morphologically as well as by their different growing behavior as adherent or suspension cells (see Section 2.5). NK-92/5.28.z cells proliferated in cocultures with target cells (Figure 4A), while NK-92 cells decreased in number (Figure 4B). At the end of the experiment, RH30 cells were almost completely lysed by NK-92/5.28.z cells, while NK-92/5.28.z cells had increased in number (Figure 4A). In contrast, NK-92 cells were not able to lyse aRMS cell monolayers, so RH30 cells overgrew the NK-92 effector cells (Figure 4B).

### 3.6. Cytotoxic Capacity of NK-92/5.28.z and Parental NK-92 Cells against aRMS Tumor Spheroids

While the 2D experiments mentioned above demonstrated the specific killing capacity of NK-92/5.28.z effector cells, in vivo, these cells would not be acting on single layers of target cells. In 3D tumors, effectors require the ability to interact physically with tumor targets as well as the ability to infiltrate their 3D structures. Moreover, 3D tumorspheres possess potentially inhibitory extracellular matrix and can accumulate anti-effector cell reactivity, and deeper layers of such 3D tumorspheres reproduce the acidity of a typical hypovascular tumor; all of these factors are critical components of an immunosuppressive TME.

**Figure 5:**
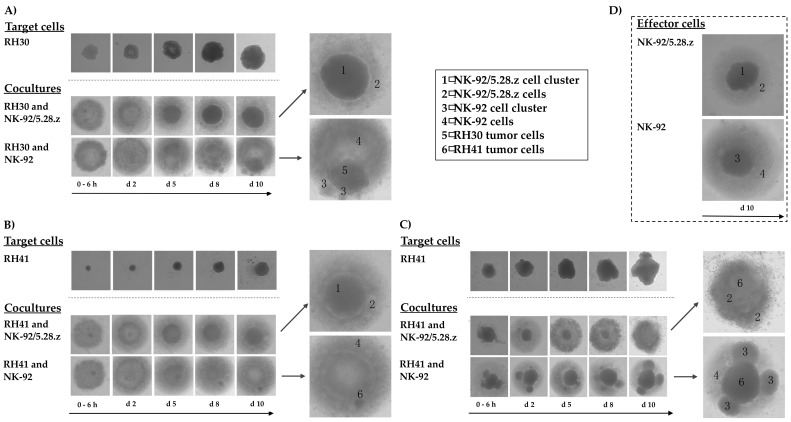
Activity of NK-92/5.28.z cells against aRMS tumor spheroids. Tumor spheroids were established from RH30 (**A**) and RH41 cells (**B**,**C**). NK-92/5.28.z or NK-92 cells were added as effector cells at an E:T ratio below 1:1, and their growing and antitumoral activity were followed over 10 days. NK-92/5.28.z cells exhibited fast and efficient lysis of aRMS tumor spheroids within two (**A**,**B**) to five days (**C**, fewer effector cells), while parental NK-92 cells were not able to efficiently lyse the tumor cells in 3D culture. Growing behaviors of target and effector cells (**D**) alone as well as in cocultures are shown in parallel, which allows for discrimination between tumor and effector cells.

To investigate the potential of NK-92/5.28.z cells to lyse aRMS cells growing in 3D culture, we established RH30 and RH41 tumor spheroids and used them to compare the killing capacity of the ERBB2-CAR-NK-92 cells with that of parental NK-92 cells over a time period of 10 days. In these experiments, NK-92/5.28.z cells were able to lyse aRMS tumor spheroids within two days of coincubation (Figure 5A,B). Furthermore, the effector cells proliferated and formed cell clusters, which is in accordance with their natural growth properties (Figure 5D). Parental NK-92 cells displayed similar growth behavior and lysed some of the tumor cells, but the remaining aRMS cells nonetheless proliferated and clustered in steadily growing tumor spheroids.

A similar outcome was observed when cocultures of RH41 cells were initiated with fewer effector cells than shown in Figure 5B; in these experiments, it took five days for NK-92/5.28.z cells to diminish the tumor cell spheroids, while parental NK-92 cells again displayed much weaker activity against the RH41 cells growing in 3D culture (Figure 5C).

### 3.7. Immunoregulatory Factors Secreted by NK-92/5.28.z and Parental NK-92 Cells in Cocultures with aRMS Cells

**Figure 6:**
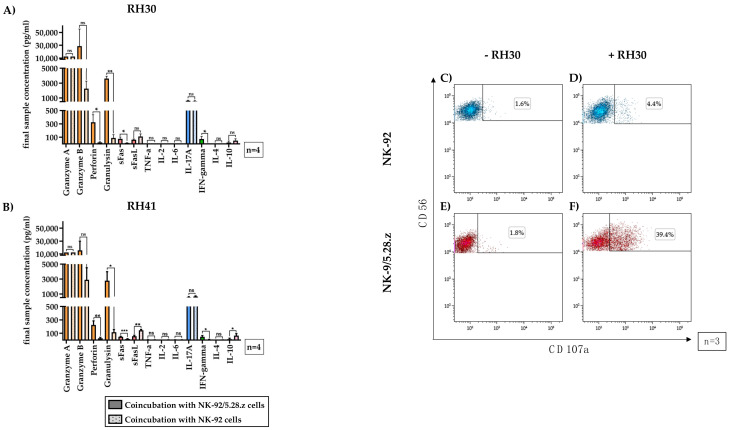
Secretion of immunoregulatory factors by activated NK-92/5.28.z cells. The supernatants of cocultures of RH30 (**A**) and RH41 (**B**) cells with NK-92/5.28.z and NK-92 cells at E:T ratios of 20:1 (absolute cell count per well: 1 × 10^5^ effector cells and 5 × 10^3^ target cells) were assessed for effector molecules and immunoregulatory factors. Mean concentrations are shown ± SD. The lytic molecules granzyme A and B, perforin and granulysin (orange bars), interleukin (IL)-17A (blue bars), interferon (IFN)-γ, and Fas ligand (FasL) (red bars) were detectable at increased concentrations in NK-92/5.28.z compared with NK-92 cocultures. Cocultures with NK-92 cells showed higher levels of FasL (red bars) and IL-10 (purple bars) than cocultures with NK-92/5.28.z cells. Tumor necrosis factor (TNF)-α, IL-2, IL-4, and IL-6 were not secreted at considerable levels. To assess their degranulation capacity, NK-92 (**C**,**D**) or NK-92/5.28.z cells (**E**,**F**) were coincubated with RH30 cells (**D**,**F**) or remained without target cells (**C**,**E**). Cells stained with CD107a and CD56 antibodies represented degranulating immune effector cells. Differences were considered significant for *p* < 0.05 (*), *p* < 0.01 (**), *p* < 0.005 (***), or not significant (ns).

Supernatants from 3-h cocultures of NK-92/5.28.z or NK-92 cells with aRMS cells (RH30, Figure 6A, and RH41, Figure 6B) were assessed for the presence of certain immunoregulatory factors using the LEGENDplex™ multianalyte immunoassay.

High levels of different effector molecules were found (Figure 6, orange bars). The mean granulysin concentrations in the NK-92/5.28.z cocultures were approximately 3.4-fold (RH30) to 4.5-fold (RH41) lower than the granzyme A concentrations, showing significant differences between NK-92/5.28.z and NK-92 cells. The mean perforin concentrations were even lower than the granulysin concentrations but remained significantly higher than the concentrations measured in NK-92 cocultures (*p* < 0.05).

The Fas receptor and its ligand (Figure 6, red bars), which facilitate apoptosis induction upon cell-cell interaction, were also investigated. Significantly more soluble Fas ligand (FasL) was seen after coincubation of aRMS cells with NK-92 cells than after coincubation of aRMS cells with NK-92/5.28.z cells.

The concentrations of tumor necrosis factor (TNF)-α, IL-2, IL-6, and IL-4 were below 10 pg/mL and therefore not quantifiable; consequently, these molecules likely had no impact on effector and target cell interactions, at least in this setting.

Coculture of either NK-92/5.28.z or NK-92 cells with aRMS cells resulted in the secretion of high concentrations of the proinflammatory cytokine IL-17A (Figure 6, blue bars). While the levels of the antitumoral cytokine interferon (IFN)-γ in cocultures with NK-92/5.28.z cells were significantly higher than those in cocultures with NK-92 cells (*p* < 0.05), the concentrations of the anti-inflammatory cytokine IL-10 (Figure 6, pink bars) in cocultures with NK-92 cells were significantly higher than those in cocultures with NK-92/5.28.z cells (*p* < 0.05). Nevertheless, IL-10 production was still measurable in NK-92/5.28.z cultures.

Degranulation capacity of immune effector cells without target cell contact remained low (basal CD107a levels for NK-92 (Figure 6C, 1.53 ± 0.33%) and NK-92/5.28.z cells (Figure 6E, 2.02 ± 0.33%) of three representative experiments are shown). After two-hour coincubation with RH30 cells, the parental NK-92 cells showed minimal enhancement of CD107a expression (Figure 6D, 4.34 ± 1.82%), while in NK-92/5.28.z cells, the CD107a expression increased to 35.65 ± 6.63% (Figure 6F).

## 4. Discussion

In this study, we employed ERBB2-CAR-modified NK-92 cells (NK-92/5.28.z), which are already available as a GMP-compliant product, as effector cells against high-risk aRMS tumor cells [25,41]. In contrast to allogeneic T cells, donor-derived CAR-NK cells or CAR effector cells generated from the NK-92 cell line can be safely administered in an allogeneic setting [23,26,46,47]. This is also the case for ERBB2-specific NK-92/5.28.z cells, which are currently being tested in a local treatment setting in a phase I clinical trial in glioblastoma patients at the University Hospital Frankfurt am Main, Germany; the treatment has so far demonstrated safety and feasibility in the single-dose dose-escalation part of this trial [21]. In preclinical models, NK-92/5.28.z cell administration is followed by the induction of tumor-specific adaptive immune responses [28,42], which suggests that these cells have dual roles as targeted killers and modulators of endogenous antitumor immunity. These dual effects might be essential for overcoming physical tumor barriers as well as the immunosuppressive TME.

The thorough preclinical characterization of NK-92/5.28.z cells in other disease indications and the available clinical experience from the ongoing trial in patients with glioblastoma may aid in the development of these cells as a treatment for other solid tumors, such as high-risk RMS, and enable more rapid preparation of a respective clinical trial [21,28,41,42]. As the first step in this direction, we performed a preclinical analysis of the cells in in vitro models of aRMS; our experiments demonstrated the potential of NK-92/5.28.z cells as adoptive immunotherapy for ERBB2-positive aRMS, justifying the further evaluation of this approach for the treatment of high-risk sarcomas in subsequent in vivo analyses as a prerequisite for a possible future phase I/II clinical trial.

ERBB2 expression has been confirmed in aRMS tumor cell lines, as recently described by Merker et al. [15], and in primary high-risk aRMS cells. The heterogeneity in cell size and morphology shown by flow cytometry (Figure 1) and adherent cultures (Figure 4) is a feature of RMS and is mostly a consequence of the residual ability of myogenic progenitor cells to differentiate along the myogenic pathway. Furthermore, in aberrant neoblastic conditions, RMS cells represent an arrested state in the development of normal skeletal muscle, showing maturation defects and giving rise to multinucleated structures [48]. This morphological pattern of RMS tumors, among others, enables their discrimination from cocultured immune effector cells in our experiments. During tumor growth, stable surface expression of ERBB2 was shown.

In addition, the expression levels of MIC-A/B and ULBP-1 on RH30 and RH41 cells were low, which is a feature previously shown to be associated with metastatic disease [24]. This finding suggests a low impact of parental NK-92 and NK-92/5.28.z cells on aRMS cells via interaction with activating NK cell receptors such as the NKG2D receptor, which likely contributed to the resistance of aRMS cells against NK-92-mediated killing seen in our study [26].

In different types of cytotoxicity assays, NK-92/5.28.z cells displayed markedly increased cytolytic activity against RMS tumor cells, including primary aRMS cells from a newly diagnosed patient, when compared to unmodified parental NK-92 cells. NK-92/5.28.z cells displayed a mean cytolytic capacity of 77.4 ± 10.8% and 61.4 ± 14.8% after three hours of coincubation with RH30 and RH41 cells, respectively, at an E:T ratio of 40:1, and this capacity increased up to mean values of 82.4 ± 11.0% and 73.3 ± 14.7% in a 16-h cytotoxicity analysis even at E:T ratios as low as 1:1. Similar killing capacity of effector cells despite differences in ERBB2 expression and number of effector cells may be explained by the excellent killing and degranulation capacities as well as by the superior proliferation behavior of NK92/5.28.z cells. The ERBB2-targeted killing of NK-92/5.28.z cells was followed by activation and rapid expansion of NK92/5.28.z cells resulting in secretion of high systemic levels of cytokines.

In fact, NK-92/5.28.z cells were able to infiltrate and efficiently lyse aRMS tumor cell monolayers as well as tumor spheroids, with the latter serving as a model for the 3D structure of tumor tissue [15]. Of note, the cytotoxicity of NK-92/5.28.z cells against aRMS cells was similar to that of NK-92/5.28.z cells against MDA-MB-453 breast cancer cells, which highly overexpress ERBB2 [30]. This suggests that CAR-engineered immune effector cells can be redirected to recognize and kill target cells even in the case of moderate target antigen expression [29,30]. Parental NK-92 cells failed to kill aRMS tumor cells; this result is in accordance with data from models of other solid tumors, which were largely resistant to unmodified parental NK-92 cells [32,41]. Furthermore, in our tumor spheroid models, which demonstrated critical components of an immunosuppressive TME, the presence of aRMS tumor cells inhibited the proliferation of NK-92 but not NK-92/5.28.z cells.

NK-92/5.28.z cells secreted high amounts of effector molecules such as granzymes, granulysin, and perforin upon interaction with RMS tumor cells, while the levels of secreted cytokines such as TNF-α, IL-2, IL-6, and IL-4 were minimal or below the detection limit. This finding is in accordance with previous findings in other solid tumor models [25,28,41]. The absence of TNF-α and IL-6 may be important in the context of safety, as both cytokines contribute to CRS, often complicating CAR-T cell therapy [49]. The secretion of IFN-γ and IL-10 may support endogenous antitumor immunity but may also modulate overactivated or dysregulated immune responses [23,25,41,50,51]. Hence, as shown in immunocompetent animal models, NK-92/5.28.z cell therapy could stimulate subsequent adaptive antitumor immunity of the host in addition to inducing direct cytotoxicity [28], which despite the fact that T cell supportive factors are often missing in solid tumors may result in stimulation of bystander immune cells that is equal to or better than that observed in a clinical trial with ERBB2-specific CAR-T cells [30].

The TME, which suppresses immune cells through hypoxic and acidic conditions, nutrient deprivation, and high amounts of immunosuppressive molecules such as TGF-β, constitutes a severe obstacle for cancer immunotherapies [31,33]. In this respect, recent findings have demonstrated that at least under controlled conditions in vitro, NK-92/5.28.z cells retain their full cytotoxic capacity in an environment similar to an inhibitory TME [42], and this maintained cytotoxicity might also be the case when encountering tumor cells in vivo.

Nevertheless, combined treatment approaches may still be necessary to increase the effectiveness of cancer immunotherapy [30,33]; for example, CAR-T cells were recently administered in combination with a checkpoint inhibitor [30,33], which may also be considered for NK-92/5.28.z cells as a multimodal antitumor approach [21].

CAR effector cells further engineered to constitutively secrete specific cytokines may also be considered in the context of maintaining or even amplifying the cytotoxic activity of CAR-immune cell therapy against resistant tumor cells [22,24,33]. In fact, cells modified to have constitutive IL-15 secretion have already shown promising results in preclinical and clinical studies [24,26,46]. Other cytokines that may be employed for combination therapies are IL-12, IL-18, and IL-24 [52,53,54]. Improved homing and tumor infiltration to further enhance the anticancer efficacy of immune effector cells may also be achieved by increased expression of chemokine receptors [28,31,33].

Nevertheless, potential on-target/off-tumor effects remain an important topic of discussion in targeted immunotherapies [27]. We and others consider ERBB2 as a suitable TAA that can be used to target high-risk RMS tumors [30], as ERBB2 expression on normal tissues is moderate and has so far not resulted in severe toxicities upon treatment of patients with CAR-T cells or NK-92/5.28.z cells, which employ the ERBB2-specific antibody FRP5 for target recognition [21,30,38]. This is in contrast to the reported death of a patient with colon cancer metastatic to lung and liver after ERBB2-CAR therapy caused by tonic signaling through a Herceptin-based third-generation CAR [55]. The cytokine storm, in addition to the high CAR-T cell dose applied, may have caused a massive activation of CAR-T cells in this case. However, due to their non-Hodgkin’s lymphoma origin, NK-92/5.28.z cells and parental NK-92 cells are irradiated prior to clinical application as a safety measure [21,22,26]. Irradiation with 10 Gy causes growth arrest of NK-92/5.28.z cells, while their cytotoxic potential is retained for 24 to 72 h [25,28,32]. Hence, repetitive infusions will be necessary for the treatment of a systemic disease such as high-risk RMS [56]. Currently, repetitive treatments with irradiated NK-92/5.28.z cells are being tested in the expansion cohort of the ongoing clinical trial in glioblastoma [21].

This is the first report of ERBB2-CAR-NK-92 cell-mediated killing of RMS cells. The results reported here demonstrate high and specific activity of NK-92/5.28.z cells against ERBB2-positive aRMS cells, justifying further efforts to develop these cells as a novel treatment approach for high-risk RMS patients. High-risk RMS patients, especially those who are older than 10 years of age, with an alveolar histopathology subtype, with unfavorable primary tumor sites, and with metastatic disease with bone or BM involvement could benefit from this approach, even if immunohistochemistry is not sufficiently sensitive to identify ERBB2 surface expression. However, ERBB2 surface expression should at least be detectable by flow cytometry with lower detection limits.

Hence, the next step will be the evaluation of the targeted NK-92 cells in preclinical mouse RMS tumor models to investigate the homing of NK-92/5.28.z cells to distant tumor sites and their elimination of residual or even bulky disease, which may be achieved by repetitive infusions [41]. For this purpose, we are envisaging a preclinical mouse model using human tumor tissue best mimicking the complexity of the high-risk features that exist in the human aRMS tumor population and that represent appropriate sites for human high-risk RMS tumors with metastatic disease, including bone or BM involvement. Hence, xenografts of the human luciferase-expressing alveolar RMS cell line RH30^GFP/Luc^, which was established from the bone marrow metastasis of a 17-year-old male patient carrying a p53 mutation and expressing the PAX3/FKHR fusion protein intravenously injected into NOD/SCID/IL-2Rγc^−/−^ (NSG) mice will be used for preclinical in vivo assessment of NK-92/5.28.z cells.

However, tumors in this xenograft mouse model do not completely mimic the human TME. In addition, mouse xenograft models are not fully useful for anticipating toxicity from targeted therapy, and in particular, interactions with the host immune system cannot be obtained. These immunological deficits can, in principle, be overcome by human immune system (HIS)-reconstituted xenograft models. These models are highly valuable but are time-consuming, expensive, and technically challenging, and therefore not suitable, especially taking into consideration the potential advantages of NK-92/5.28.z cells as a potent, universal, and cost-effective off-the-shelf product and the urgent need to deploy this cellular therapeutic for the treatment of high-risk RMS.

## 5. Conclusions

Herein, we investigated ERBB2-specific NK-92 cells (NK-92/5.28.z) as a well-defined, third-party, off-the-shelf CAR-engineered cell product to target high-risk RMS cells. The efficacy of NK-92/5.28.z cells against aRMS tumor cells growing in 2D and 3D culture was extensively tested and compared to that of parental NK-92 cells, revealing high and specific cytotoxicity of the CAR-engineered but not the unmodified NK cells against rhabdomyosarcoma cells in addition to the secretion of high levels of proinflammatory cytokines and cytotoxic effector molecules. Taken together, these data justify further efforts to develop NK-92/5.28.z cells as a novel treatment approach for patients with ERBB2-positive high-risk RMS. The next step will be the evaluation of the targeted NK-92 cells in preclinical mouse RMS tumor models to investigate the homing of NK-92/5.28.z cells to distant tumor sites and their elimination of residual or even bulky disease as a prerequisite for subsequent evaluation in a clinical trial.

## Data Availability

The data presented in this study are openly available or are available on request from the corresponding author.

## Supplementary Materials

The following are available online at https://www.mdpi.com/2072-6694/13/6/1443/s1. Figure S1: Video Analysis of Target Cell Killing of NK-92/5.28.z and Parental NK-92 Cells by Time-lapse Microscopy; Figure S2: Chimeric antigen receptor expression.
