# Supplementary figures and images for: Retargeting of NK-92 Cells against High-Risk Rhabdomyosarcomas by Means of an ERBB2 (HER2/Neu)-Specific Chimeric Antigen Receptor
Source: Cancers (Basel). 2021 Mar 22;13(6):1443. doi: 10.3390/cancers13061443 (PMC8004684; doi:10.3390/cancers13061443)

# ERBB2-CAR Expression

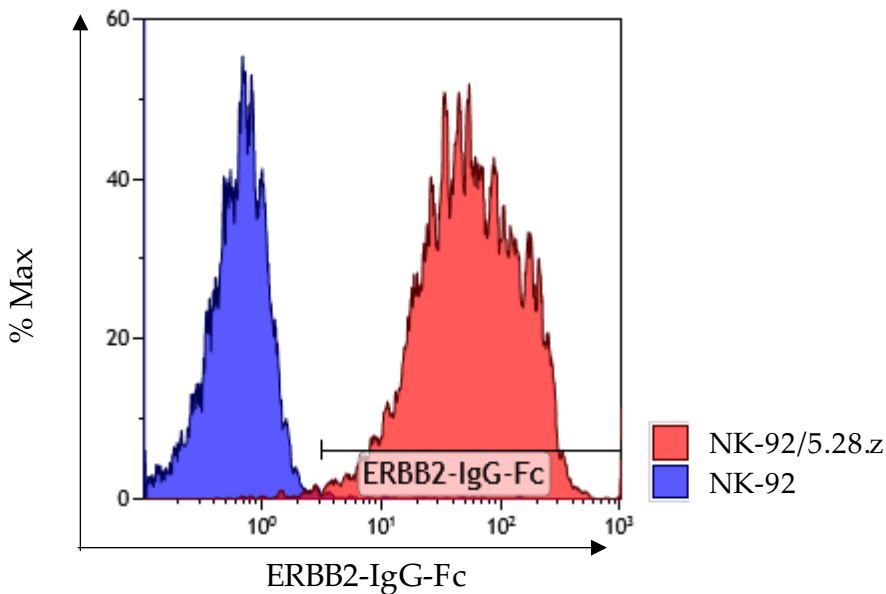

Supplement: Supplementary file 1 [file cancers-13-01443-s001.pdf]
